# Synthesis and Characterization of π-SnS Nanoparticles and Corresponding Thin Films

**DOI:** 10.3390/nano11030767

**Published:** 2021-03-18

**Authors:** Sreedevi Gedi, Vasudeva Reddy Minnam Reddy, Salh Alhammadi, Hyeonwook Park, Chelim Jang, Chinho Park, Woo Kyoung Kim

**Affiliations:** School of Chemical Engineering, Yeungnam University, Gyeongsan, Gyeongbuk 38541, Korea; drsrvi9@gmail.com (S.G.); drmvasudr9@gmail.com (V.R.M.R.); salehalhammadi.1987@gmail.com (S.A.); greatekal@naver.com (H.P.); jclaa1541@naver.com (C.J.); chpark@ynu.ac.kr (C.P.)

**Keywords:** nanoparticles, thin films, π-SnS, cubic tin sulfide, precursor concentration, photovoltaic, hydrogen production

## Abstract

Tin sulfide polymorph (π-SnS) nanoparticles exhibit promising optoelectrical characteristics for photovoltaic and hydrogen production performance, mainly because of the possibility of tuning their properties by adjusting the synthesis conditions. This study demonstrates a chemical approach to synthesize π-SnS nanoparticles and the engineering of their properties by altering the Sn precursor concentration (from 0.04 M to 0.20 M). X-ray diffraction and Raman studies confirmed the presence of pure cubic SnS phase nanoparticles with good crystallinity. SEM images indicated the group of cloudy shaped grains, and XPS results confirmed the presence of Sn and S in the synthesized nanoparticles. Optical studies revealed that the estimated energy bandgap values of the as-synthesized π-SnS nanoparticles varied from 1.52 to 1.68 eV. This work highlights the effects of the Sn precursor concentration on the properties of the π-SnS nanoparticles and describes the bandgap engineering process. Optimized π-SnS nanoparticles were used to deposit nanocrystalline π-SnS thin films using the drop-casting technique, and their physical properties were improved by annealing (300 °C for 2 h).

## 1. Introduction

Semiconducting metal chalcogenide nanoparticles have attracted research attention owing to their tunable absorption properties for photovoltaic and hydrogen production applications [1,2,3,4,5,6]. In particular, there has been an increasing demand for tin sulfide (SnS) nanoparticles in recent years as photovoltaic materials [3], lithium battery anode materials, and photocatalytic materials owing to their favorable optoelectrical properties, which can be easily altered by manipulating the synthesis conditions [7].

Until now, orthorhombic SnS (α-SnS) was one of the most widely reported inorganic binary thin film solar cell materials, with a record efficiency (η) of 4.36% reported in 2014 [8]. For this, α-SnS was prepared by atomic layer deposition followed by subsequent annealing in an H_2_S atmosphere, which enhances the production cost. In addition, the α-SnS thin film solar cell showed a low open-circuit voltage (V_OC_) of 0.372 V [9]. In 2015 [10], the polymorph of tin sulfide, chemically synthesized cubic SnS (π-SnS) with a large band gap of ~1.7 eV, was reported. This cubic structure is identical to that of SnS nanoparticles or nanocrystals [11] but can produce a higher V_OC_ due to its wider bandgap property. Chemically fabricated π-SnS thin film solar cell with the structure of stainless steel (SS)/π-SnS /CdS/ZnO/ZnO:Al/Ag showed η of 1.28% with a relatively higher V_OC_ of 0.470 eV compared to the α-SnS based solar cell [10]. On the other hand, metal monochalcogenides (SiS, SiSe, SiTe, GeS, GeSe, GeTe, SnS, and SnSe) were theoretically predicted to be economical and eco-friendly hydrogen production photocatalysts [12,13,14]. The polymorph of tin sulfide, π-SnS exhibited interesting characteristics such as a high absorption coefficient of 10^−4^ cm^−1^ in the visible region, a direct optical bandgap of 1.7 eV, and a p-type conducting nature with dark conductivity of about 10^−6^ Ω^−1^ cm^−1^ [9], and had good photoconductivity response [10]. In addition, both constituent elements of Sn and S are earth-abundant, inexpensive, and non-toxic. According to the literature, α-SnS has been explored for photocatalytic activity [15], but π-SnS was hardly studied despite of its promising characteristics.

The properties of nanoparticles are highly dependent on the method of synthesis and preparative conditions such as precursor nature and concentration, synthesis temperature and time, solution pH, and the nature of the chelating agent [16]. To exploit the novel properties of nanoparticles for a variety of applications, it is essential to tune the size, phase, structure, shape, and composition by optimizing the above-mentioned parameters.

In this study, we report the synthesis of π-SnS nanoparticles using a simple chemical precipitation (CP) method. The influence of the Sn precursor concentration on the structural, morphological, compositional, and optical properties was studied, and the Sn precursor concentration was optimized for the synthesis of pure and high-quality π-SnS nanoparticles. In addition, π-SnS thin films were prepared using drop-casting technique and heat treatment was applied to enhance their quality for photovoltaic and hydrogen production applications.

## 2. Materials and Methods

### 2.1. Materials

The analytical reagents employed in this study were tin (II) chloride (SnCl_2_·2H_2_O, Sigma-Aldrich), thioacetamide (C_2_H_5_NS, Sigma-Aldrich, St. Louis, MO, USA), ethylenediaminetetraacetic acid (EDTA, C_10_H_16_N_2_O_8,_ Sigma-Aldrich, St. Louis, USA), and acetone (C_3_H_6_O, Sigma-Aldrich, St. Louis, USA), used as received without further purification and stored in a humidity-controlled desiccator cabinet. For the preparation of π-SnS nanoparticles, SnCl_2_·2H_2_O and C_2_H_5_NS were used as Sn and S sources, respectively, and C_10_H_16_N_2_O_8_ was used as a chelating agent.

### 2.2. Experimental Details

**Figure 1:**
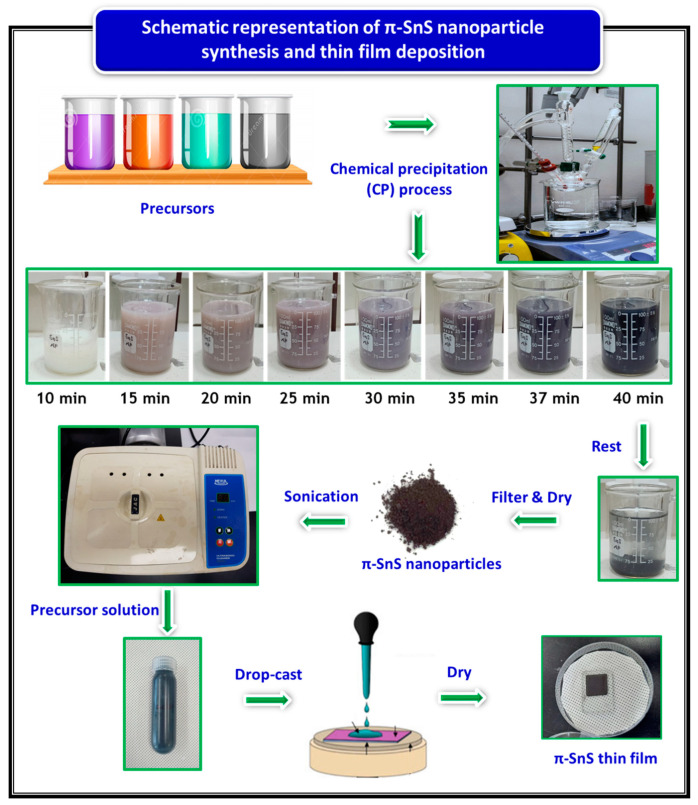
Schematic representation of tin sulfide polymorph (π-SnS) nanoparticles synthesis and π-SnS thin film preparation.

The π-SnS nanoparticles were synthesized using a simple chemical precipitation (CP) process. In a standard procedure, a 0.5 M C_2_H_5_NS solution and SnCl_2_·2H_2_O solution at a selected concentration (0.04, 0.08, 0.12, 0.16, and 0.20 M corresponding to [Sn]/[S] ratio of 0.08, 0.16, 0.24, 0.32, and 0.40) were prepared using deionized water (DI) water and acetone, respectively. The prepared SnCl_2_·2H_2_O precursor solution along with 0.05 M EDTA chelating agent were poured into a 100 mL beaker and stirred vigorously for 10 min at 40 °C under an ambient atmosphere until a clear solution was obtained, which indicated the formation of complex tin ions through a reaction process. Next, the C_2_H_5_NS precursor solution was added into the reaction mixture, which resulted in a systematic color change of the solution from white to black during the next 40 min. Throughout the reaction, the stirring speed was maintained at 250 rpm, and the reaction solution was cooled to room temperature after completion of the process. The schematic representation of π-SnS nanoparticles synthesis and thin film preparation is shown in Figure 1. The precipitates collected from the solution were washed thoroughly using DI water before centrifugation to remove residual impurities. The product was then dried at 110 °C for 6 h in an oven. The powder was collected in a vial and placed in a desiccator for characterization.

### 2.3. Characterization Details

The powder X-ray diffraction (XRD) patterns were recorded on a Seifert 3003TT X-ray diffractometer (Almelo, The Netherlands) with CuKα radiation (λ = 1.5405 Å). Raman analysis was performed using a Horiba Jobin-Yvon Lab Ram HR 800 spectrometer (Tokyo, Japan) at room temperature. The morphology was studied using a Hitachi S-4800 scanning electron microscope (Tokyo, Japan) coupled with energy-dispersive X-ray spectroscopy (EDS) and X-ray photoelectron spectroscopy (XPS:K-Alpha, Thermo Fisher Scientific, Dartford, UK), respectively. Optical reflectance spectra were recorded using a Cary 5000 UV-Vis-NIR spectrophotometer (Santa Clara, CA, USA).

## 3. Results and Discussion

### 3.1. Growth of π-SnS Nanoparticles

The growth of π-SnS nanoparticles by the CP process involves the following steps: (1) formation of a tin complex from the tin source and the chelating agent, (2) reduction of Sn^2+^ from the tin complex, (2) reduction of S^2−^ from the sulfur source, and (3) a precipitation reaction between Sn^2+^ and S^2−^, resulting in the formation of π-SnS nanoparticles.

In the first stage, the Sn^2+^ ions from tin (II) chloride are complexed by the chelating agent, ethylenediaminetetraacetic acid, as follows:

SnCl_2_∙2H_2_O + C_10_H_16_N_2_O_8_ ⇌ Sn(C_10_H_16_N_2_O_8_)^2+^ + 2Cl^−^ + 2H_2_O

In a regulated manner, the complex ions start to release free Sn^2+^ ions. Therefore, when the tin complex dissociates

Sn(C_10_H_16_N_2_O_8_)^2+^ ⇌ Sn^2+^ + C_10_H_16_N_2_O_8_

the concentration of complex tin ions [Sn(L)^2+^ or Sn(L)^4+^] in the solution can be controlled by adjusting the concentration of the Sn precursor or the complexing agent [17].

The generation of sulfur ions from the S precursor in the solution is also an essential factor during the process. Hydrolysis of the S precursor produces S^2−^ ions by the following reactions [18]:

CH_3_CSNH_2_ + H_2_O ⇌ CH_3_CONH_2_ + H_2_S

When the reaction attains an equilibrium condition [19], the following reactions are expected:

H_2_S + H_2_O ⇌ H_3_O^+^ + HS^−^

HS^−^ ⇌ H^+^ + S^2−^

HS^−^ + OH^−^ ⇌ H_2_O + S^2−^

The precipitation reaction between Sn^2+^ and S^2−^ results in the formation of π-SnS nanoparticles by the following equation [20]:

Sn^2+^ + S^2−^ → SnS

### 3.2. Properties of π-SnS Nanoparticles

(a) XRD results

**Figure 2:**
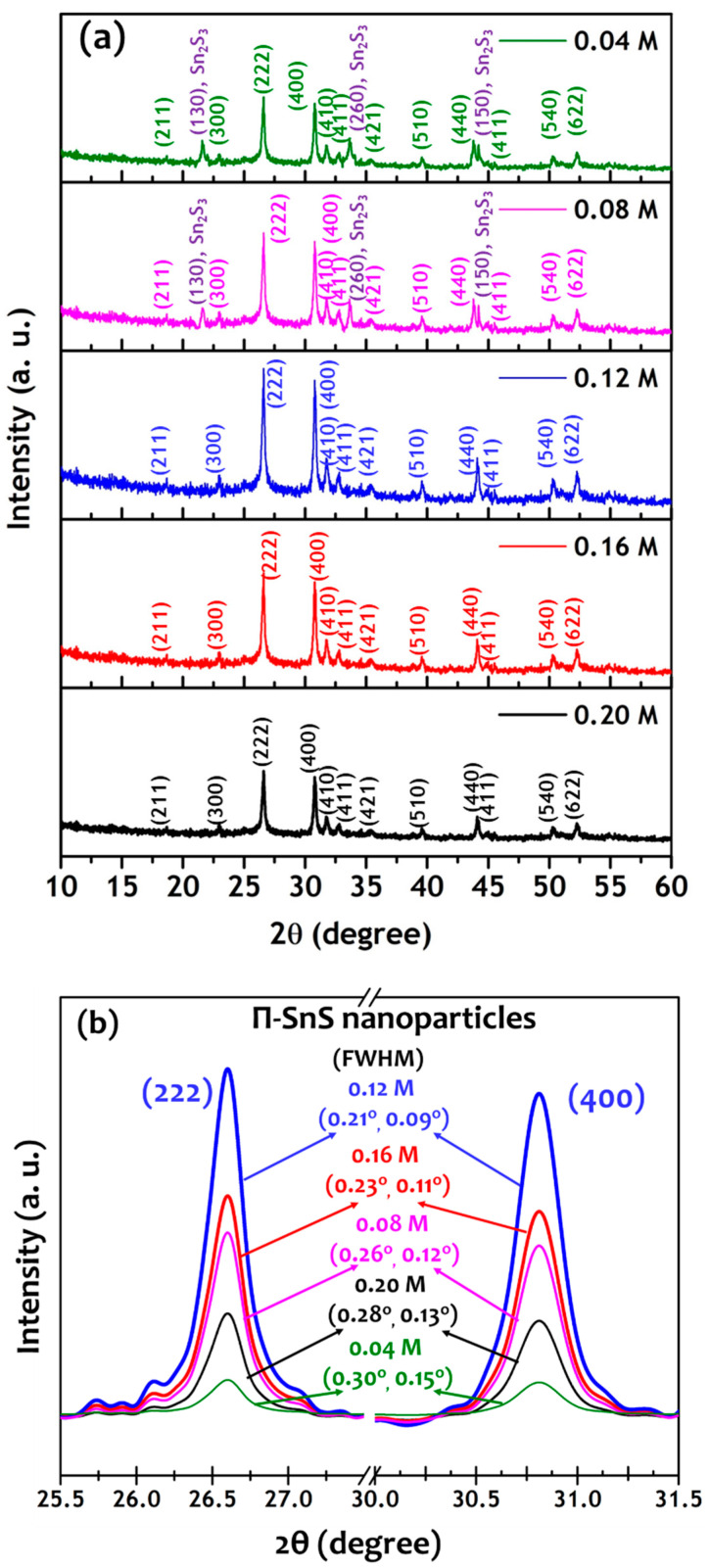
(**a**) XRD diffraction patterns and (**b**) magnified (222) and (400) peaks with full width at half maximum (FWHM) of π-SnS nanoparticles synthesized by different Sn precursor concentration of 0.04, 0.08, 0.12, 0.16 and 0.20 M corresponding to [Sn]/[S] ratio of 0.08, 0.16, 0.24, 0.32, and 0.40, respectively.

Figure 2 shows XRD patterns of the as-synthesized π-SnS nanoparticles. The XRD patterns of the particles synthesized with Sn precursor concentrations of 0.04–0.20 M show intense diffraction at 2θ angles of 26.6° and 30.8°, in addition to weak diffractions at 18.6°, 23.0°, 31.7°, 32.7°, 35.6°, 39.5°, 44.1°, 44.9°, 50.3°, and 52.2° related to the (222) and (400) planes, and (211), (300), (410), (411), (421), (510), (440), (441), (540), and (622) planes, respectively. All the observed planes correspond to the cubic structure of SnS (π-SnS) with the P2_1_3 space group [21,22]. However, the π-SnS nanoparticles synthesized with low Sn precursor concentrations of 0.04 M and 0.08 M showed additional diffraction peaks related to Sn_2_S_3_ phase. As the Sn precursor concentration increased from 0.12 M to 0.20 M, the amplitude of all the diffraction peaks decreased and the full width at half maximum (FWHM) values of both (222) and (400) peaks increased. The π-SnS nanoparticles synthesized at a Sn precursor concentration of 0.12 M attained a maximum amplitude with the smallest FWHM of 0.21° and 0.09° for (222) and (400) peaks, respectively, as shown in Figure 2b. The lattice parameter of the π-SnS nanoparticles synthesized at a Sn precursor concentration of 0.12 M was found to be a = 11.59 Å, which is perfectly correlated with the lattice parameter for the cubic unit cell of SnS [23].

(b) Raman results

**Figure 3:**
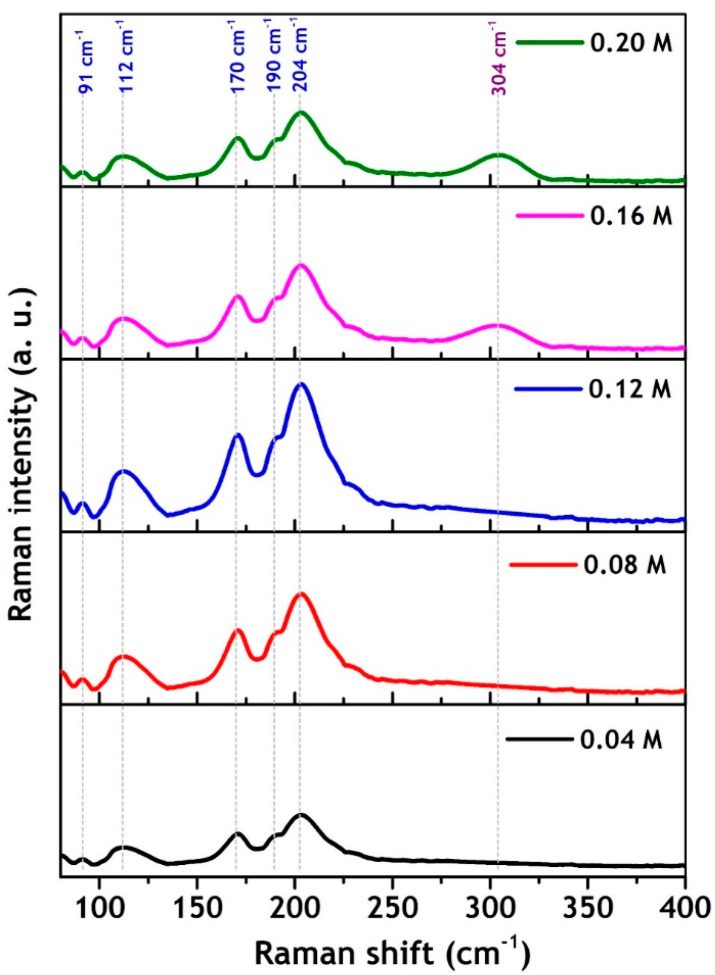
Raman spectra of as-synthesized π-SnS nanoparticles synthesized by different. Sn precursor concentration of 0.04, 0.08, 0.12, 0.16, and 0.20 M.

Raman analysis was carried out to further confirm the phase purity of the as-synthesized nanoparticles, and the corresponding spectra are shown in Figure 3. The Raman spectra of the nanoparticles synthesized at different Sn precursor concentrations varying from 0.04 M to 0.12 M show two prominent phonon modes at 170 cm^−1^ and 204 cm^−1^, along with a group of weak modes (91 cm^−1^, 112 cm^−1^, and 190 cm^−1^) within the ranges 89–125 cm^−1^ and 180–230 cm^−1^, corresponding to characteristic Raman modes of the cubic SnS phase [23,24]. While at low Sn precursor concentrations of 0.04 M and 0.08 M, the Raman spectra of the nanoparticles also shows a slight hump at 304 cm^−1^, related to the characteristic phonon mode of Sn_2_S_3_ [25,26], revealing that the low Sn precursor concentration promotes the development of a sulfur-rich secondary phase, in agreement with the XRD results.

(c) SEM results

**Figure 4:**
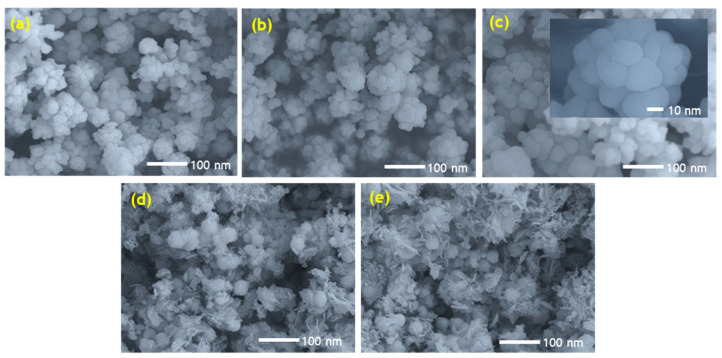
SEM images of as-synthesized π-SnS nanoparticles with different Sn precursor concentrations of (**a**) 0.20 M, (**b**) 0.16 M, (**c**) 0.12 M, (**d**) 0.08 M, and (**e**) 0.04 M.

Figure 4a–e show SEM images of the as-synthesized π-SnS nanoparticles. SEM images of π-SnS nanoparticles synthesized with lower Sn precursor concentrations (0.04 M and 0.08 M) show cloud-shaped grains fully covered with thin flower-like petals, whereas the SEM images of π-SnS nanoparticles synthesized with Sn precursor concentrations varied from 0.12 M to 0.20 M show groups of similar cloud-shaped grains with uniform shape, which indicates the impact of Sn precursor concentration on the morphology of the nanoparticles. The slight difference in the morphology of nanoparticles at low Sn precursor concentrations (<0.12 M) could be due to the presence of an additional tin sulfide phase, as confirmed by both XRD and Raman spectroscopy. The SEM images also reveal that the size of the as-synthesized nanoparticles is in the range 26–45 nm.

(d) EDAX and XPS results

**Figure 5:**
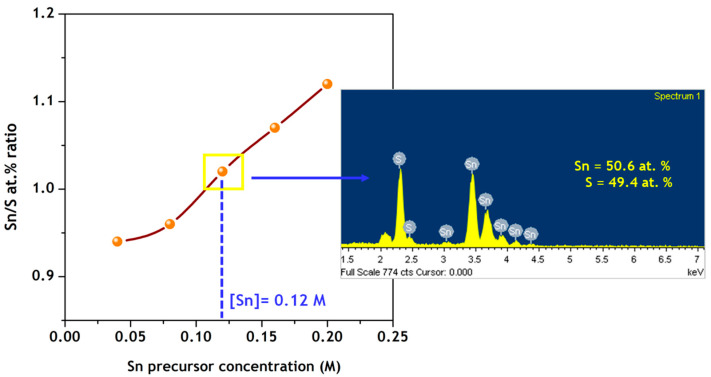
Variation of elemental composition ratio as-synthesized π-SnS nanoparticles.

The chemical composition of nanoparticles synthesized with different Sn precursor concentrations varied in the range 0.04–0.20 M was analyzed by EDAX. The analysis indicated that nanoparticles synthesized at all Sn precursor concentrations show Sn and S peaks, which confirms that Sn and S elements are both present in the nanoparticles. The variation in the Sn/S atomic ratio of the as-synthesized nanoparticles with respect to the Sn precursor concentration is shown in Figure 5. The Sn/S atomic ratio increases from 0.94 to 1.12 with an increase of concentration from 0.04 M to 0.20 M, indicating that Sn is more dominant at higher concentration values. However, the nanoparticles synthesized at a Sn precursor concentration of 0.12 M show an atomic ratio of 1.02, indicating that the nanoparticles have a precise stoichiometry between the constituent elements of Sn and S.

**Figure 6:**
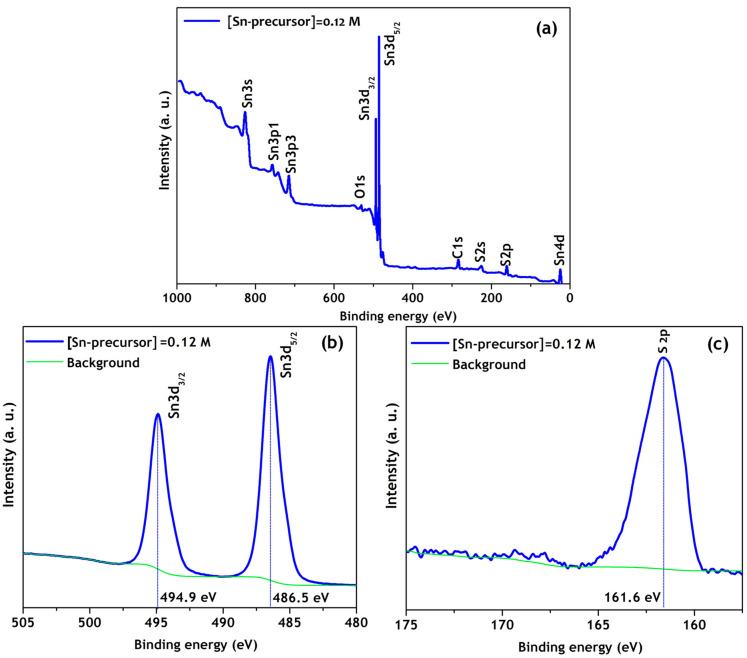
(**a**) XPS survey spectrum, and high-resolution spectra for (**b**) Sn 3d and (**c**) S 2p of π-SnS nanoparticles synthesized at a Sn precursor concentration of 0.12 M.

The XPS analysis was also performed on π-SnS nanoparticles synthesized at a Sn precursor concentration of 0.12 M. Figure 6 shows the XPS survey spectrum in the binding energy range of 0–1000 eV, along with XPS core-level spectra of Sn and S. The existence of Sn and S elements in the nanoparticles was confirmed by the full scan XPS spectrum (Figure 6a). The small O-and C-related peaks were presumably due to ambient contamination and reference, respectively. In addition, there were no other elemental impurities in the synthesized nanoparticles. The core-level XPS spectrum of Sn in Figure 6b shows Sn 3d doublet at 486.5 eV and 494.9 eV corresponding to the Sn 3d_5/2_ and Sn 3d_3/2_ energy levels, respectively, with a separation of 8.4 eV. The core-level XPS spectrum of S in Figure 6c displays S 2p binding energy of 161.6 eV, related to the S^2−^–Sn^2+^ bonding, which confirmed the presence of SnS in π-SnS nanoparticles synthesized at a Sn precursor concentration of 0.12 M. Moreover, the composition of π-SnS nanoparticles was found to be 50.2 at.% and 49.1 at.% for Sn and S, respectively.

(e) Optical studies

**Figure 7:**
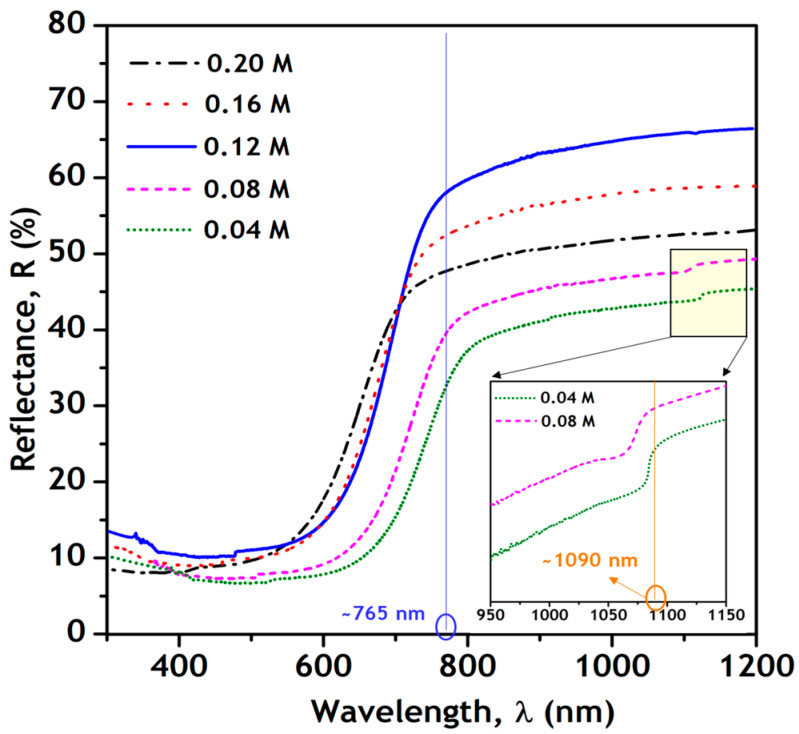
Reflectance spectra of as-synthesized π-SnS nanoparticles.

**Figure 8:**
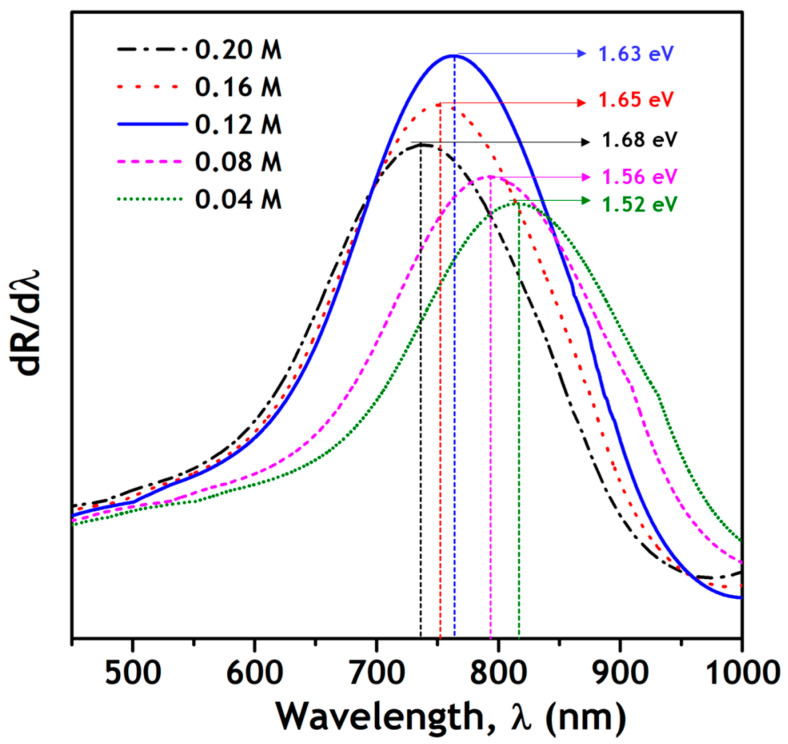
Plots of differential reflectance spectra (dR/dλ vs. λ) of as-synthesized π-SnS nanoparticles.

Figure 7 shows the reflectance spectra of the as-synthesized nanoparticles recorded in the range 300–1200 nm. The spectra of π-SnS nanoparticles synthesized with Sn precursor concentrations of 0.04 M to 0.12 M show a sharp drop at the absorption edge (~765 nm). In contrast, the nanoparticles synthesized with a concentration of 0.16 M and 0.20 M show a second absorption edge near 1090 nm, confirming the presence of an additional tin sulfide phase (Sn_2_S_3_) in the nanoparticles. The bandgap of the π-SnS nanoparticles was also determined from the differential reflectance spectra (dR/dλ vs. λ), as shown in Figure 8. The steep absorption edge in the reflectance spectra results in a hump in the plot of dR/dλ vs. λ, and a Gaussian fit of the hump gives the bandgap of the nanoparticles [27]. The obtained bandgap values of the as-synthesized π-SnS nanoparticles varied from 1.52 to 1.68 eV. The π-SnS nanoparticles synthesized with a Sn precursor concentration of 0.12 M showed an optical bandgap of 1.62 eV. The bandgap values obtained in this study directly correlate with the values reported in literature [9].

### 3.3. Properties of π-SnS Thin Films

(a) Deposition process

Thin film deposition is carried out using a nanoparticle ink dispersed in a solvent with the help of sonication. The nanoparticle ink is spread onto the specified substrate (generally glass or Mo and FTO or ITO for photovoltaic and hydrogen production applications, respectively) to form thin films using various deposition processes, such as spin coating, spraying, dip coating, screen printing, drop-casting, or by doctor blade. The π-SnS nanoparticles synthesized at a Sn precursor concentration of 0.12 M were dispersed in chloroform at a concentration of ~5 mg/mL and sonicated to form a uniform π-SnS nanoparticle ink. π-SnS thin films were prepared on a Mo substrate (2.5 × 2.5 cm) using the drop-casting technique, and the entire process is shown in the schematic diagram (Figure 1). The samples were then dried in air at 65 °C on a hot plate for 1 min to eliminate the remaining solvent. The thickness of the deposited thin film could be precisely monitored and replicated through the repeated coating and drying processes. For effective light absorption, a thickness of ~1 μm was used. The prepared π-SnS thin film was annealed at 300 °C for 2 h, and the physical properties of the nanocrystalline π-SnS thin films were examined using suitable characterization methods, as defined in Section 2.3.

(b) Physical properties

**Figure 9:**
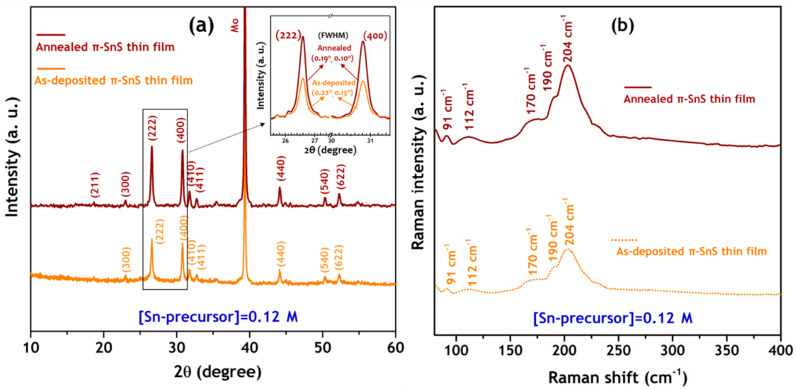
(**a**) XRD patterns and (**b**) Raman spectra of as-deposited and annealed π-SnS thin films, prepared by π-SnS nanoparticles synthesized at a Sn precursor concentration of 0.12 M.

Figure 9a shows the XRD patterns of the as-deposited and annealed thin films. The XRD patterns of the as-deposited thin film exhibit peaks related to the cubic structure of SnS (π-SnS). In the case of the annealed thin film, the amplitude of all diffraction peaks is strengthened presumably due to further grain growth by thermal annealing. As shown in the inset plot of Figure 9a, the FWHM values for (222) and (400) peaks were decreased from 0.22° and 0.13° to 0.19° and 0.10°, respectively, after annealing, which supported grain growth. The peak at 2θ = 40.50° in the XRD spectra is related to the (110) plane of Mo coated on the substrate [28], representing an increase in crystallinity after annealing. No impurities such as binary tin sulfides (SnS_2_, Sn_2_S_3,_ etc.) or oxides (SnO_2_) are detected in the XRD patterns. Figure 9b shows the Raman spectra of the as-deposited and annealed thin films. Both films display two characteristic Raman modes at 170 cm^−1^ and 204 cm^−1^, ascribed to the cubic structure of SnS. The Raman mode findings are in good agreement with previous reports [11,24]. The amplitude of the phonon modes increases for the annealed film, indicating an increase in the crystallinity of the films and a decrease in defects.

**Figure 10:**
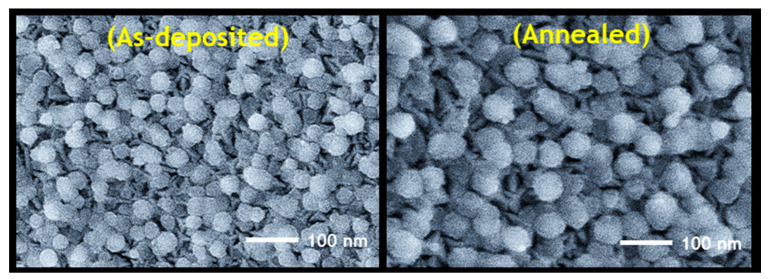
SEM surface images of as-deposited and annealed π-SnS thin films, prepared by π-SnS nanoparticles synthesized at a Sn precursor concentration of 0.12 M.

**Figure 11:**
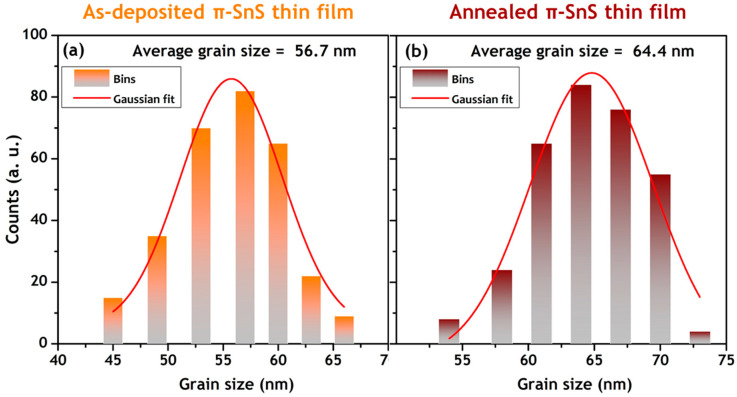
Gaussian grain size distribution of (**a**) as-deposited (**b**) annealed π-SnS thin films.

Figure 10 shows the surface morphology of the as-prepared and annealed π-SnS thin films. The SEM images show that both films have spherical uniform grains with a compact morphology and no noticeable holes or cracks. The Gaussian grain size distribution of the as-prepared and annealed π-SnS thin films is shown in Figure 11a, b, respectively. The plots show that the as-deposited π-SnS thin film has slightly smaller grains (~56 nm) than the annealed π-SnS thin film (~64 nm). Thus, the annealing of the π-SnS thin film improved the grain quality and grain size, suggesting that the grains obtained enough thermal energy to enable effective film growth. The same trend in grain growth with annealing temperature has also been reported previously [7].

**Figure 12:**
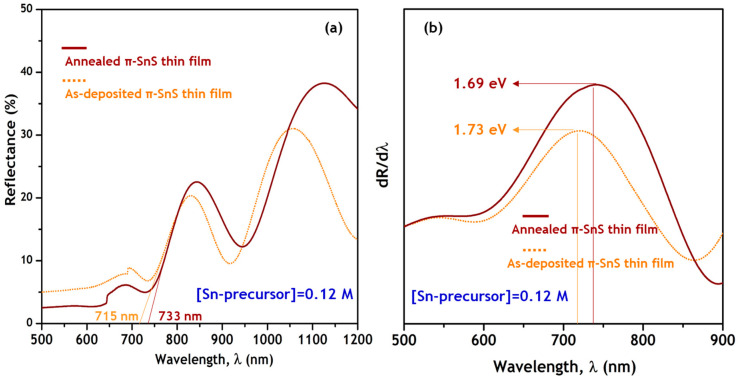
(**a**) Optical reflectance spectra and (**b**) plots of dR/dλ vs. λ of as-deposited and annealed π-SnS thin films.

Figure 12a displays the diffuse reflectance spectra of the as-prepared and annealed π-SnS thin films recorded over the wavelength range 500–1200 nm. The average optical reflectance of the as-prepared film was approximately 28%, whereas that of the annealed film was 36% above the fundamental absorption edge. The as-deposited π-SnS thin film shows an absorption edge near 715 nm, which is red-shifted to 733 nm for the annealed π-SnS thin film, because of an improvement in the crystallinity and surface morphology of the films during the annealing process.

The bandgap of the films was determined from the differential reflectance spectra (dR/dλ vs. λ) displayed in Figure 12b. The as-prepared and annealed π-SnS thin films produced the band gaps of nearly 1.73 eV and 1.69 eV, respectively. The difference in the bandgap of the π-SnS thin film after the annealing treatment is attributed to an improvement in the quality and size of grains with the heat treatment [29]. The obtained direct energy gap values agree well with previously reported data [9,30].

## 4. Conclusions

In this study, new polymorphs of tin sulfide, cubic SnS (π-SnS) nanoparticles, were synthesized using a simple chemical precipitation (CP) process by varying the Sn precursor concentration from 0.04 M to 0.20 M. The influence of the Sn precursor concentration on the structural, morphological, compositional, and optical properties was studied using appropriate characterization tools. XRD studies revealed that the as-synthesized π-SnS nanoparticles have cubic crystalline nature. EDS analysis revealed the presence of Sn and S in the synthesized nanoparticles. SEM analysis showed that the synthesized nanoparticles are grouped in cloud-shaped grains, and the morphology changed if the Sn precursor concentration increased beyond 0.12 M. The obtained optical bandgap energies varied from 1.52 to 1.68 eV. Thus, the results show that the π-SnS nanoparticles synthesized with the Sn precursor concentration of 0.12 M showed good crystallinity, stoichiometry, and morphology. Then, π-SnS thin films were prepared using optimized π-SnS nanoparticles by the drop-casting technique and annealed at 300 °C for 2 h. The annealing treatment enhanced the crystallinity and morphology of the π-SnS thin films.

## Data Availability

The data is available on reasonable request from the corresponding author.
